# A Simple and Reliable Dispersive Liquid-Liquid Microextraction with Smartphone-Based Digital Images for Determination of Carbaryl Residues in *Andrographis paniculata* Herbal Medicines Using Simple Peroxidase Extract from *Senna siamea* Lam. Bark

**DOI:** 10.3390/molecules27103261

**Published:** 2022-05-19

**Authors:** Sam-ang Supharoek, Watsaka Siriangkhawut, Kate Grudpan, Kraingkrai Ponhong

**Affiliations:** 1 Department of Chemistry and Center of Excellence for Innovation in Chemistry, Faculty of Science, Mahidol University, Bangkok 10400, Thailand; samang.sup@mahidol.ac.th; 2 Department of Medical Science, Mahidol University, Amnatcharoen Campus, Amnat Charoen 37000, Thailand; 3 Creative Chemistry and Innovation Research Unit, Department of Chemistry and Center of Excellence for Innovation in Chemistry, Faculty of Science, Mahasarakham University, Maha Sarakham 44150, Thailand; watsaka@hotmail.com; 4 Department of Chemistry, Faculty of Science and Center of Excellence for Innovation in Analytical Science and Technology for Biodiversity-Based Economic and Society, Chiang Mai University, Chiang Mai 50200, Thailand; kgrudpan@gmail.com

**Keywords:** carbaryl, cassia bark (*Senna siamea* Lam.), smartphone-based digital image analysis, 1-naphthol, peroxidase enzyme

## Abstract

A simple and reliable dispersive liquid-liquid microextraction (DLLME) coupled with smartphone-based digital images using crude peroxidase extracts from cassia bark (*Senna siamea* Lam.) was proposed to determine carbaryl residues in *Andrographis paniculata* herbal medicines. The method was based on the reaction of 1-naphthol (hydrolysis of carbaryl) with 4-aminoantipyrine (4-AP) in the presence of hydrogen peroxide, using peroxidase enzyme simple extracts from cassia bark as biocatalysts under pH 6.0. The red product, after preconcentration by DLLME using dichloromethane as extraction solvent, was measured for blue intensity by daily life smartphone-based digital image analysis. Under optimized conditions, good linearity of the calibration graph was found at 0.10–0.50 mg·L^−1^ (*r^2^* = 0.9932). Limits of detection (LOD) (3SD/slope) and quantification (LOQ) (10SD/slope) were 0.03 and 0.09 mg·L^−1^, respectively, with a precision of less than 5%. Accuracy of the proposed method as percentage recovery gave satisfactory results. The proposed method was successfully applied to analyze carbaryl in *Andrographis paniculata* herbal medicines. Results agreed well with values obtained from the HPLC-UV method at 95% confidence level. This was simple, convenient, reliable, cost-effective and traceable as an alternative method for the determination of carbaryl.

## 1. Introduction

Since the outbreak of the COVID-19 virus in 2019, the application of medicinal plants to strengthen immunity and prevent viral infections has increased as another option [1,2,3]. *Andrographis paniculata* is an indigenous medicinal plant found in Malaysia and Thailand [1,2,4,5] that has antioxidant properties to scavenge free radicals [6,7] and stimulate the immune system [8,9] against foreign matter entering the body, thereby inhibiting the growth of cancer cells [5,10,11,12]. This plant is widely used for treating sore throats, flu and upper respiratory tract infections [5,13]. Therefore, the use of pesticides increases the harvest of *Andrographis paniculate* [14].

Carbaryl (1-naphthyl methylcarbamate), a synthetic insecticide in the carbamate family, causes reproductive and developmental toxicity including neurodevelopmental perturbations that impact the immune system as a possible human carcinogen. Carbaryl exhibits high toxicity to non-target organisms [15] but is only moderately toxic to aquatic organisms (LC50 values in rainbow trout and bluegill of 1.3 and 10 mg·L^−1^, respectively) [16]. Oral LD50 of carbaryl ranges from 250 mg·kg^−1^ to 850 mg·kg^−1^ in rats, and from 100 mg·kg^−1^ to 650 mg· kg^−1^ in mice [17].

Various analytical methods have been established for the determination of carbaryl such as high-performance liquid chromatography (HPLC) [18,19,20], liquid chromatography-mass spectrometry (LC-MS) [21,22], liquid chromatography-tandem mass spectrometry (LC-MS/MS) [23], gas chromatography (GC) [24,25] and capillary electrophoresis [26,27]. These techniques can simultaneously determine carbaryl with other carbamate insecticides but they require highly skilled operators and are cost-consuming. Electrochemical analysis [28,29,30,31] to investigate carbaryl content has advantages in terms of specific and low detection limits. However, this method requires skilled fabrication and modification of the working electrode. Fluorescence spectroscopy has also been used to determine carbaryl [32,33], providing advantages of selectivity detection, while spectrophotometric methods [34,35,36,37,38] are based on carbaryl hydrolysis to 1-naphthol, which is subsequently coupled with different reagents. Spectrophotometric procedures are considered to be appropriate as they involve commonly available laboratory instruments such as a spectrometer but they still need some specific requirements [39,40].

Recently, image processing was applied for chemical analysis. Digital image acquisition devices such as a scanner, camera and smartphone built-in camera as cheap electronic components were employed to capture digital photo images [39]. Digital imaging analysis is an advanced technique to evaluate the color intensity of captured digital photo images using image processing software [41]. Pixels in red, green or blue channels with numerical values ranging from 0 to 255 can be utilized for analytical calibration [42]. Digital images provide data that can be used for fast and low-cost colorimetric detection of quantitative chemical analysis [43]. Smartphones are extensively employed in daily life as image acquisition tools because they are portable, with multiple data transmission functions and high storage capacity [44]. Digital image data acquired from image software can considerably enhance detection accuracy [45,46]. Digital image analysis has been applied in various fields including environmental monitoring, food safety and clinical analysis due to its convenience, stability, low cost and flexibility [47,48]. Digital image analysis has also been developed to determine liquid turbidity [42] and bacterial cell concentration in liquid media [49].

Enzymes are often employed as bio-accelerators because they require small amounts of substrate and utilize specific reactions that operate under mild conditions [50,51]. Peroxidase enzymatic spectrophotometry has been used to quantify carbaryl residues in vegetables by our group [52,53]. Cassia bark (*Senna simea*), an indigenous plant found in many areas including Thailand, Southern India, Sri Lanka, Myanmar (Burma), Cambodia, Malaysia and parts of Indonesia was employed as a source of peroxidase enzyme for extraction by a simple method yielding crude peroxidase enzyme. Crude peroxidase enzyme extract was used as an alternative to commercially available expensive horseradish peroxidase as a biocatalyst for the enzymatic reaction of carbaryl with 4-AP in the presence of hydrogen peroxide under optimal pH. No additional enzyme purification steps were required to avoid deterioration through enzyme degradation [54,55,56]. This process offered comparable analytical performance to horseradish peroxidase as a green chemical analysis.

However, a sample containing carbaryl at low levels in complicated matrices requires pretreatment before the colorimetric detection step. Sample pretreatment techniques such as dispersive liquid-liquid microextraction (DLLME) have recently attracted increased attention to concentrate the sample before analysis [52,57,58]. DLLME has advantages of simplicity of operation, rapidity, low cost, high recovery, high preconcentration factor and environmental benignity [59].

Here, we developed a simple downscaling cost effective procedure as a green chemical analysis for the colorimetric enzymatic determination of carbaryl residues in herbal medicine samples by employing crude peroxidase enzyme extracts with smartphone-based digital image analysis, using dispersive liquid-liquid microextraction for sample pretreatment.

Here, low-cost crude peroxidase enzyme extract from cassia bark was employed as biocatalysts. A down-scaled dispersive liquid-liquid microextraction technique reduced the consumption of deleterious extraction and disperser solvent to preconcentrate the analyte before daily life smartphone-based digital imaging analysis. The developed method was simple, cost-effective, reliable and tracible and produced comparable results to HPLC.

## 2. Results and Discussion

### 2.1. Activity of Peroxidase Crude-Extract

Peroxidase enzyme extracts from cassia bark were mixed with ready-to-use ABTS substrate in the presence of hydrogen peroxide. The green color of ABTS cation radical was observed, indicating that peroxidase was found in the crude extracts from cassia bark. Activity of the crude enzyme was 1.15 ± 0.05 kU 10 μL^−1^, equivalent to 0.32 kU L^−1^ of horseradish peroxidase (HRP, Sigma-Aldrich, St. Louis, MO, USA) evaluated from HRP activity calibration in the range 0.125–2.0 kU L^−1^ [52]. Peroxide activity remained constant for 3 months at −20 °C. Activity decreased by 9% after 6 months compared with fresh extracts, while the stability of enzyme extracts declined after 8 h at room temperature, indicating that they could be used for quantification of carbaryl for only one day.

### 2.2. Suggested Reaction between Peroxidase Extracts and Carbaryl

**Figure 1:**
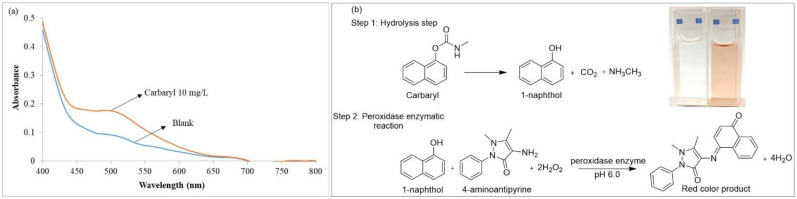
Determination of carbaryl based on peroxidase enzymatic reaction; (**a**) Absorption spectra 10 mg·L^−1^ carbaryl in phosphate buffer pH 6.0 and blank; (**b**) suggested mechanism for the reaction of carbaryl with 4-AP in the presence of hydrogen peroxide, exploiting crude peroxidase as a catalyst.

The suggested reaction for the determination of carbaryl was based on the mixed solution containing 1-naphthol as the hydrolysis product of carbaryl under alkaline conditions, 4-AP and hydrogen peroxide. The mixture solution was catalyzed by crude peroxidase enzyme extracts under phosphate buffer pH 6. Maximum absorption of red product was observed at a wavelength of 500 nm (Figure 1a).

Figure 1b shows the reaction involving two steps as (i) hydrolysis of carbaryl under alkaline conditions yielding 1-naphthol, and (ii) enzymatic reaction of 1-naphthol with 4-AP in the presence of hydrogen peroxide using peroxidase as a catalyst. 4-AP reacted with 1-naphthol at the para-position of the aromatic ring giving a red-colored product [60]. Results indicated that carbaryl insecticide could be produced using crude peroxidase enzyme extracts from cassia bark.

### 2.3. Optimization of Operational Parameters for Determination of Carbaryl by Smartphone-Based Digital Images

A light-emitting diode (LED) (Yongnuo YN300 III, China) was placed in the middle of the light control box used as the illumination device. This eliminated the need for flash photography to capture images and control the uniformity of light intensity (Figure S1). The captured images of the 96-microwell plate without extraction phase and the repeatability of RGB intensities were used to assess the homogeneity of light illumination from the LEDs. Relative standard deviation (RSD) was 1.47%, indicating excellent measurement repeatability of RGB intensity under the light control box. The RSD obtained from the calibration graph in the range 0.10 to 0.50 mg·L^−1^ of standard carbaryl was less than 6% (*n* = 9). We concluded that light and temperature in a closed light control box were not significantly affected by the intensity of RGB color.

Image qualities obtained from the camera and smartphone were not significantly different. The RGB intensity of captured images using standard carbaryl at 0.30 mg·L^−1^ achieved using a smartphone and camera were not significantly different (t Stat = 0.123 < t Critical = 1.985 at 95% confidence level and df = 95). Moreover, the slope of the calibration graph obtained from the smartphone was not significantly different when compared with the slope of the calibration curve from the camera (t Stat = 1.14 < t Critical = 2.78 at 95% confidence level and df = 4).

After enzymatic reaction and DLLME optimization, the extraction solvent was transferred to a 96-microwell plate to capture the digital image under the light control box using a smartphone. RGB intensity of the photographed image was evaluated by ImageJ software to obtain the RGB profile as illustrated in Figure S2. For quantification purposes, the graph of RGB intensity difference (ΔI) (difference in color intensity of reagent blank zone and color intensity of standard) was plotted against carbaryl concentrations in the range 0 to 0.50 mg·L^−1^ (Figure S3). Blue (B) and green (G) intensity of the captured image related to the concentration of carbaryl but blue intensity gave the highest sensitivity. Therefore, blue intensity was utilized to determine carbaryl by the proposed method.

Sensitivity of the developed method depended on the volume of extraction phase in the microwell plate and was investigated at 100, 200 and 300 μL. The loading extraction phase at 200 μL gave good sensitivity and linearity for quantification of carbaryl. Low sensitivity was achieved at 100 μL, while 300 μL gave a narrow linear range and low sensitivity. Hence, 200 μL of the extraction phase was loaded into the 96-microwell plate. The calibration graph was constructed by plotting the change in intensity of blue color (intensity of blank − intensity of analyte) versus carbaryl concentration ranging from 0.10 to 0.50 mg·L^−1^.

### 2.4. Optimized Conditions for Determination of Carbaryl Using Crude Peroxidase Enzyme

#### 2.4.1. Effect of pH

**Figure 2:**
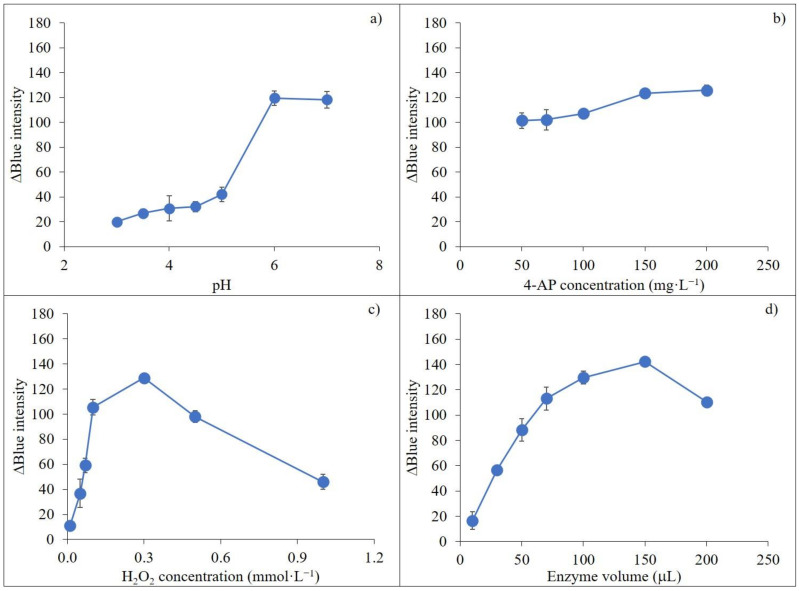
Investigation of various carbaryl detection parameters based on peroxidase enzymatic reaction; (**a**) effect of pH; (**b**) effect of 4-AP concentration; (**c**) effect of hydrogen peroxide concentration and (**d**) effect of enzyme volume on sensitivity of carbaryl detection by peroxidase enzymatic reaction.

The pH value impacts stability, conformation and activity of an enzyme. The effect of pH on the enzymatic reaction for carbaryl assay was determined using citrate-phosphate buffer (pH 3 to pH 5) and phosphate buffer (pH 6 to pH 7). Results in Figure 2a show that crude peroxidase extract provided the highest sensitivity using phosphate buffer pH 6. Therefore, the peroxidase enzyme catalytic reaction for carbaryl assay was operated at pH 6.0.

#### 2.4.2. Effect of 4-AP Concentration

4-AP chromogenic substrate was employed for peroxidase enzymatic reaction to determine carbaryl. 4-AP acts as a hydrogen atom donor in the peroxidase catalytic reaction. Concentrations of 4-AP from 50 to 200 mg·L^−1^ were investigated. Results indicated that the analytical signal increased gradually with increasing 4-AP concentration from 50 to 150 mg·L^−1^. The signal was not significantly different from 150 to 200 mg·L^−1^ because active sites of peroxidase were almost saturated with 4-AP (Figure 2b). Therefore, 150 mg·L^−1^ of 4-AP was selected for the next experiment.

#### 2.4.3. Effect of Hydrogen Peroxide Concentration

Hydrogen peroxide acts as a hydrogen atom acceptor. Results in Figure 2c show that the signal increased sharply between 0.01 and 0.3 mmol·L^−1^ hydrogen peroxide and then dropped until 1.0 mmol·L^−1^ due to hydrogen peroxide inhibition, with 0.3 mmol·L^−1^ providing the highest value. This concentration was selected for subsequent experiments.

#### 2.4.4. Effect of Peroxidase Enzyme Volume

The volume of peroxidase enzyme extracts impacted sensitivity by influencing enzyme activity. Here, 10–200 μL of enzyme extracts were tested. Enzyme activity increased with increasing volume of enzyme, accelerating the reaction. When increasing the volume of enzyme, the red color of the blank also increased. Results are presented in Figure 2d. The signal increased gradually from 10 μL to 150 μL and then decreased above 150 μL. Thus, 150 μL volume was selected for subsequent experiments.

#### 2.4.5. Effect of Incubation Time

Longer incubation time increased products from the catalytic reaction. Incubation time ranging 1–20 min was studied. Sensitivity climbed continuously from 1 min to 10 min and then the signal leveled off over 10 min (data not shown). Therefore, 10 min incubation time was chosen for the next procedure giving sufficient determination sensitivity and short time of analysis.

### 2.5. DLLME Optimization for Carbaryl Detection

#### 2.5.1. Effect of Types and Volume of Extraction Solvents

**Figure 3:**
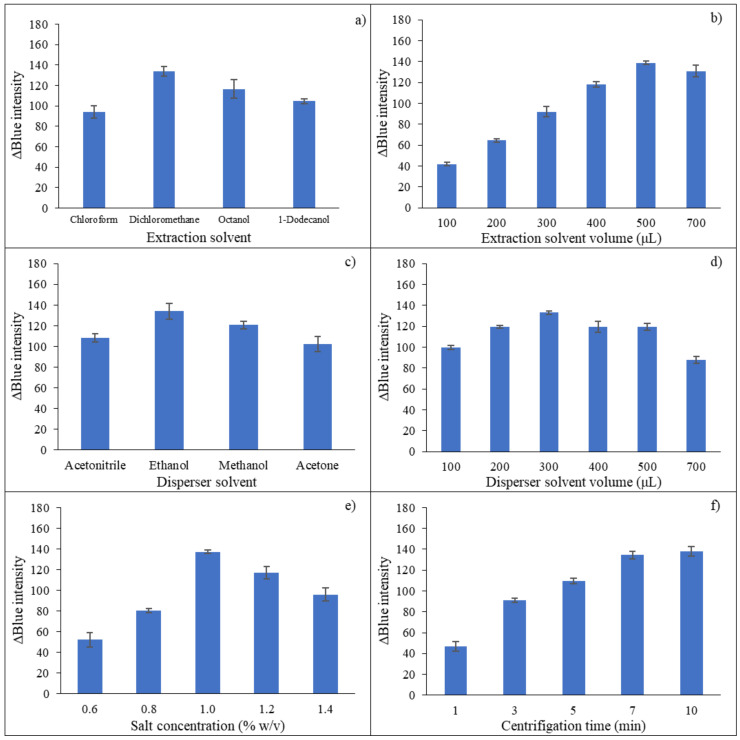
Investigation of DLLME parameters on carbaryl detection using smartphone-based digital image analysis; (**a**) effect of extraction solvent type; (**b**) effect of extraction solvent volume; (**c**) effect of dispersive solvent type; (**d**) effect of dispersive solvent volume; (**e**) effect of salt concentration and (**f**) effect of vortex time on the sensitivity of carbaryl determination.

Types and volume of extraction solvents in DLLME impacted extraction efficiency [57,59]. Chloroform, dichloromethane, octanol and 1-dodecanol were explored, with results shown in Figure 3a. Optimal extraction efficiency for carbaryl was obtained when dichloromethane was used as the extraction solvent, and this was selected for the proposed method. Volumes of 100–700 μL extract solvent were also considered. Results showed that the signal increased from 100 μL to 500 μL and then decreased from 500 μL to 700 μL because of the dilution effect (Figure 3b). Thus, 500 μL of extraction solvent was selected for subsequent experiments.

#### 2.5.2. Effect of Types and Volume of Dispersive Solvents

Dispersive solvents must have a good tendency between organic (extraction solvent) and aqueous phases and should be selected according to the miscibility properties of the extraction solvent and aqueous phase [61,62]. Different dispersive solvents such as acetonitrile, ethanol, methanol and acetone were investigated. Ethanol provided the highest signal compared to the other tested solvents (Figure 3c) and was selected as the dispersive solvent of DLLME. To evaluate the effect of dispersive solvent volume on extraction efficiency, a constant volume extraction solvent (dichloromethane at 500 µL) containing different volumes of ethanol from 100 µL to 700 µL was studied for the DLLME process. The signal at 300 µL ethanol showed maximum sensitivity (Figure 3d).

#### 2.5.3. Effect of Ionic Strength

Sodium chloride was added to improve extraction efficiency through the salting-out effect by decreasing the solubility of the analyte [52,63]. Results are shown in Figure 3e. The signal sharply increased from 0.6 to 1.0% (*w*/*v*) and then reduced from 1.2 to 1.4% (*w*/*v*). Thus, 1.0% (*w*/*v*) sodium chloride was chosen for the developed method.

#### 2.5.4. Effect of Vortex Time

Vortex mixing increases contact between the extraction solvent and the analyte to improve extraction efficiency. The analysis signal increased from 0.1 min to 1 min (data not shown), while at over 1 min the signal leveled off because equilibrium was attained. Therefore, 1 min vortex time was adopted.

#### 2.5.5. Effect of Centrifugation Time

Centrifugation to complete phase separation between the organic and aqueous phases was examined in the range 1–10 min at 4032 g. Results showed that the signal climbed continuously from 1 to 7 min, with little increase up to 10 min (Figure 3f). Thus, centrifugation at 7 min was selected to achieve phase separation for the proposed method.

### 2.6. Analytical Characteristics

**Figure 4:**
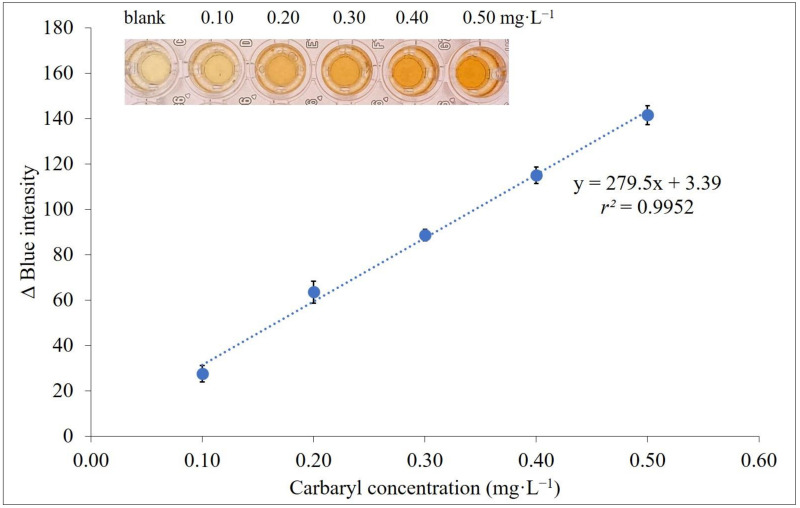
Calibration graph for carbaryl determination using smartphone detection plots between Δblue intensity and carbaryl concentrations in the range 0.10 to 0.50 mg·L^−1^.

Under optimal conditions of the proposed procedure summarized in Table S1, the linearity range of the calibration graph with DLLME for digital images based on the colorimetric method was 0.10 to 0.50 mg·L^−1^ carbaryl with good linear regression *r^2^* at more than 0.99 (Figure 4). LOD and LOQ were calculated by 3SD/slope and 10SD/slope, where SD is the standard deviation of the blank, at 0.03 and 0.09 mg·L^−1^, respectively. Precision of the digital image method when analyzing carbaryl intraday at 0.30 mg·L^−1^ (*n* = 7) was 4.91%, with reproducibility 7.59% for 7 days (*n* = 3 × 7). The calibration graph was prepared daily to minimize inherent experiment variability when using crude peroxidase enzyme extracts. A summary of the analytical characteristics of the proposed method compared to some other spectrophotometric methods/digital image colorimetry is shown in Table 1. The developed method was sensitive and simple as an alternative for the determination of carbaryl. The developed procedure was down-scaled into a microliter volume operation using 500 µL of extraction and dispersive solvent (300 μL) for the DLLME procedure compared with the previous peroxidase enzymatic spectrophotometry [52], while exploiting daily life smartphones as acquisition tools provided rapidity and traceability for carbaryl detection.

### 2.7. Recovery and Carbaryl Residues in Andrographis paniculata Herbal Medicines

Recovery of the developed method was studied by adding standard 0.10, 0.20 and 0.30 mg·L^−1^ carbaryl into real samples, with results presented in Table 2. Carbaryl sample recovery ranged 83–109% and 88–114% for smartphone-based digital images and HPLC-UV, respectively. RSDs of recovery were less than 10% indicating that the method showed good accuracy and good precision.

This method was also applied for carbaryl determination in 10 *Andrographis paniculate* herbal medicines. Carbaryl contents shown in Table 2 ranged 5.54 ± 0.13–16.22 ± 0.29 and 6.31 ± 0.70–15.56 ± 0.32 mg·kg^−1^ for smartphone-based digital images and HPLC, respectively. Concentrations of carbaryl residues in *Andrographis paniculate* herbal medicines obtained from both methods were not significantly different at 95% confidence level using the paired *t*-test (t Stat = 1.06, t Critical = 2.57, df = 5). High contamination of carbaryl in *Andrographis paniculate* herbal medicines was observed in sample Nos. 1, 4–7 and 10. There are no reports regarding maximum residue limit (MRL) of carbaryl in herbal medicines.

### 2.8. Selectivity for the Determination of Carbaryl by Peroxidase Enzymatic Reaction

Other insecticides in the carbamate family such as carbofuran, promecarb, aldicarb and isoprocarb were studied for the selectivity of the peroxidase enzyme catalytic reaction. Only carbaryl was catalyzed by peroxidase enzyme, while carbofuran, promecarb, aldicarb and isoprocarb were not observed [52,53]. Therefore, the quantification of carbaryl using peroxides enzyme extracts from cassia bark was selective since the other carbamates did not interfere with carbaryl detection.

## 3. Materials and Methods

### 3.1. Reagents and Chemicals

All chemicals used were analytical grade and utilized without further purification. Deionized (DI) water (Milli-Q, Millipore, Solna, Sweden) was used to prepare all the solutions, while 1000 mg·L^−1^ carbaryl stock solution (Sigma-Aldrich, Darmstadt, Germany) was prepared by weighing 0.10 g carbaryl, with volume adjusted to 100 mL with 95% ethanol (Merck, Darmstadt, Germany). Working solutions of carbaryl were freshly prepared by appropriate dilution of carbaryl stock solution by deionized water. Hydrogen peroxide 100 mmol·L^−1^ was prepared by transferring 1.02 mL 30% hydrogen peroxide solution into a 100 mL volumetric flask and adjusting the volume with deionized water. 4-AP, 1000 mg·L^−1^, was prepared by weighing 0.10 g of 4-AP in a 100 mL volumetric flask and adjusting the volume with deionized water. Buffer solutions were prepared by mixing appropriate volumes of disodium hydrogen phosphate and citric acid, with the required pH attained by adjusting with sodium hydroxide solution.

### 3.2. Instruments and Apparatus

A UV-Visible spectrophotometer (UV-1800 Shimadzu, Kyoto, Japan) was utilized to evaluate enzyme activity at a wavelength of 420 nm. A Rotanta 46 R model centrifuge (Hettich Zentrifugen, Tuttlingen, Germany) was employed to achieve separation of extract solutions, yielding a clear supernatant. A pH meter (Eutech, Ayer Rajah Crescent, Singapore) was used to measure buffer pH. Reaction temperature was controlled by a water bath (Memmert, Schwabach, Germany). A vortex mixer was used to increase mass transfer of QuEChERS (Quick Easy Cheap Effective Rugged Safe) and DLLME steps. A cooking blender model EBR 2601 from Electrolux (Electrolux, Bangkok, Thailand) was utilized to homogenize the materials. iPhone model 11 Pro Max (Designed by Apple in California Assembled in China) was utilized to photograph the color products after preconcentration by DLLME under the light control box.

### 3.3. Light Control Box

The in-house light control box was adapted from our previous project [64] and constructed from white opaque acrylic sheet with outer dimensions 19 × 32 × 15 cm to prevent light penetration from the surroundings (Figure S1). The outer part was covered with PVC sticker sheet. A tray to place a 96-microwell plate was installed in the middle of the box. Internal illumination was provided by a LED video light with 300 high quality LED light beads of extra-large luminous chips (Yongnuo YN300 III, Shenzhen, China). An ON/OFF switch was used to control the power supply (6.5–8.5 V, 3 A). the LED light was positioned below the acrylic tray in the box used as a light diffuser. A hole 3.0 × 3.5 cm (w·l) was made in the top of the box for photography using a daily life smartphone built-in camera (iPhone 11 Pro Max, Apple, Zhengzhou, China).

### 3.4. Extraction of Peroxidase Enzyme from Cassia Bark

Fresh cassia bark (200 g) was collected from Amnat Charoen Province, washed with deionized water and cut into small pieces. Next, 200 g of cassia bark was weighed into a 600 mL beaker. Phosphate buffer extracted solution 100 mL, pH 6.0 was then added. The mixed contents were thoroughly blended for 5 min. The solution was then filtered using a white cloth and centrifuged at 3028× *g*, 4 °C for 30 min and 9072× *g*, 4 °C for 30 min. The supernatant was filtered using Whatman No. 1 filter paper and stored in a brown 1.5 mL microcentrifuge tube at −20 °C.

### 3.5. Peroxidase Enzyme Extract Activity Study

Peroxidase enzyme activity, not previously studied in cassia bark, was investigated to confirm the presence of the enzyme in the extract solution [52]. Briefly, 10 μL crude extract solution was mixed with specific peroxidase enzyme substrate ready-to-use ABTS solution (ABTS solution, Roche, Mannheim, Germany). Absorbance was immediately monitored at 420 nm for 1 min, and the initial slope of the enzymatic reaction was calculated. Enzyme activity of 1 U was defined as the amount of enzyme required to generate 0.001 absorbance of product every minute under the described conditions.

### 3.6. Peroxidase Enzymatic Analytical Method Synergied with DLLME for Determination of Carbaryl by Smartphone-Based Digital Image Analysis

**Figure 5:**
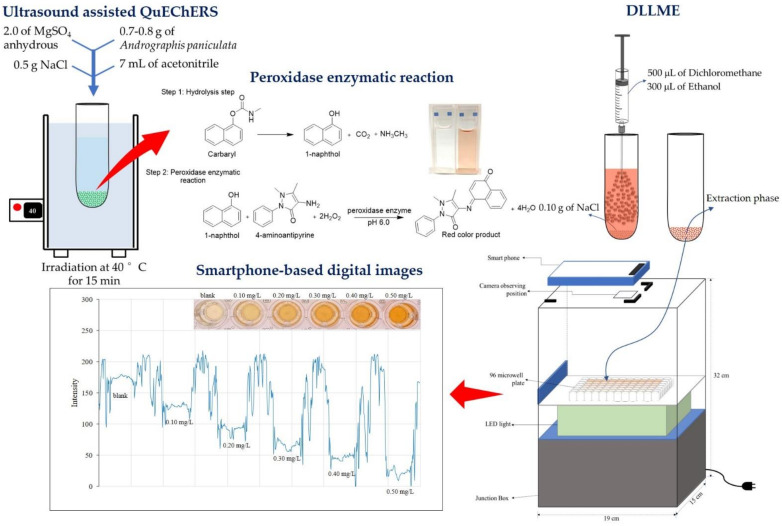
Illustration of DLLME synergy with smartphone-based digital images for the determination of carbaryl using enzymatic reaction of crude peroxidase enzyme extracts from cassia bark as biocatalyst.

As illustrated in Figure 5, samples (200 µL, see Section 3.10), NaOH (100 μL, 50 mmol·L^−1^), 4-AP (1.5 mL, 1000 mg·L^−1^), hydrogen peroxide (300 µL, 10 mmol·L^−1^) were transferred into a 10 mL volumetric flask. Then, the enzyme extract (150 µL) was added before adjusting the volume with phosphate buffer (pH 6.0, 50 mmol·L^−1^). The mixture was incubated at 30 °C for 15 min until a red color product was observed. The mixture was then transferred to a 15 mL centrifuge tube containing sodium chloride (0.10 g) and dichloromethane (500 µL, as extraction solvent), and rapidly injected with ethanol (300 µL, as disperser solvent) before vortexing for 1 min, followed by 7 min centrifuging (4032 g). The aqueous phase was completely withdrawn using a long needle syringe and the organic phase was diluted with 95% ethanol (500 μL). An aliquot (200 µL) of the resulting solution was transferred into a microwell plate and photographed using a smartphone in a light control box (Figure S1). Image processing was performed for RGB (red, green, blue) intensity of the captured image. The calibration graph was a plot of ΔB intensity against carbaryl concentration ranging 0.10 to 0.50 mg·L^−1^.

### 3.7. Optimization of Carbaryl Determination Conditions Using Crude Peroxidase Enzyme

The influence of various parameters including pH, 4-AP concentration, hydrogen peroxide concentration, peroxidase crude enzyme volume and incubation time were investigated to determine the optimized conditions. The effect of pH was studied in the range 3–6, with 4-AP concentration at 50–200 mmol·L^−1^. Hydrogen peroxide concentration was examined between 0.01 and 1 mmol·L^−1^, while crude peroxide enzyme volumes were determined in the range 10–200 μL. Incubation time of 1–20 min was also studied.

### 3.8. Optimization for DLLME

Various parameters influence the DLLME procedure. Optimized conditions for DLLME were investigated for extraction solvent type, extraction solvent volume, dispersive solvent type, dispersive solvent volume, salt concentration, vortex time and centrifuge time to enhance sensitivity before the smartphone-based digital image determination step. Extraction solvent types as chloroform, dichloromethane, octanol and 1-dodecanol were employed. The effect of extraction solvent volume was explored at 100–700 μL. Dispersive solvent type was studied for acetonitrile, methanol, ethanol and acetone. Volume of dispersive solvent was explored in the range 100–700 μL. Salting out of NaCl during the DLLME process was determined between 0.6 and 1.4% (*w*/*v*) NaCl concentration. Vortex time was also tested between 0.1 and 2 min, while the influence of centrifuge time was investigated in the range 1–10 min.

### 3.9. Validation Methods

To evaluate method validation, linearity range, limit of detection (LOD), limit of quantification (LOQ), precision in terms of relative standard deviation (RSD) and accuracy were investigated. A linear calibration graph was studied by varying carbaryl standard in the range 0.10–0.50 mg·L^−1^ under the selected conditions. A calibration curve was constructed by plotting blue intensity difference (*Y*-axis) versus carbaryl concentration in mg·L^−1^ (*X*-axis), with the linear equation and linear regression coefficient (*r^2^*) also evaluated. The precision of the proposed method was studied by monitoring the blue intensity of carbaryl standard at 0.10 and 0.30 mg·L^−1^ for seven replicates intraday (repeatability) and repeated for five days (reproducibility). Precision of the proposed method was reported in terms of RSD. The accuracy of this method was researched by adding various concentrations of 0.10, 0.20 and 0.30 mg·L^−1^ of carbaryl standard solution to the sample and evaluating the recovery percentage of carbaryl under optimal conditions. LOD and LOQ were calculated from 3SD/slope and 10SD/slope, respectively where SD represents the standard deviation of the blank.

### 3.10. Samples

Pharmaceutical preparations containing *Andrographis paniculata* were collected from different drug stores in Maha Sarakham Province, Thailand. Samples were extracted by the ultrasonication-assisted QuEChERs method [53]. Briefly, *Andrographis paniculata* powder in the capsules was weighed at 0.7–0.8 g into a 15 mL centrifuge tube. Then, 2.0 of MgSO_4_ anhydrous and 0.5 g NaCl were added, followed by pipetting 7 mL of acetonitrile extraction solvent. The solution mixture was homogenized by a vortex mixer for 1 min and then transferred to an ultrasonic bath for irradiation at 40 °C for 15 min. The sample was then centrifuged at 4032× *g* for 10 min to separate the solid and organic extraction solvent. The extraction phase was filtered through Whatman filter paper No. 1 into a 10 mL volumetric flask and the volume was adjusted with acetonitrile. Next, 3 mL of the extraction phase was transferred to a 15 mL centrifuge tube containing 10 mg of activated charcoal powder for color elimination. The solution was then vortexed for 1 min and then centrifuged at 1008× *g* for 10 min. The organic extraction phase was filtered through a nylon filter, 0.45 µm, and kept at −4 °C until analysis via peroxidase enzymatic reaction and followed by DLLME.

### 3.11. Reference Method

HPLC-UV detection was proposed as the reference method to compare sample concentrations of carbaryl. Chromatographic separation was conducted using a Waters 1525 HPLC System with a Binary Pump (Waters, Milford, MA, USA) and a LiChroCART^®^ 150-4.6 RP-18 endcapped (4.6 × 150 mm, 5.0 μm) column (Merck, Darmstadt, Germany). An isocratic system involving 40% *v*/*v* acetonitrile in deionized water with a flow rate of 1.0 mL·min^−1^ was performed. The samples were injected manually using a Rheodyne injector with a 20 μL sample loop. Absorption of carbaryl was detected at 270 nm using a Waters 2489 UV detector (Waters, Milford, MA, USA). Breeze software version 2.0 was adopted for data acquisition and peak area integration.

## 4. Conclusions

A simple and reliable dispersive liquid-liquid microextraction with smartphone-based digital images for determination of carbaryl residues was developed. A simple peroxidase extract from *Senna siamea* Lam. bark served as a catalyst for the reactions at pH 6 of 4-aminoantipyrine, hydrogen peroxide and 1-naphthol, which was the hydrolysis product of carbaryl. Dispersive liquid-liquid microextraction was synergized with peroxidase enzymatic reaction to pre-concentrate the analyte. The red color product was sensed by a smartphone camera for further evaluation to quantify the carbaryl content. The developed procedure, with micro-liter volume operation, was applied for carbaryl residue assay in *Andrographis paniculata* herbal medicine. Results were not significantly different from the HPLC-UV reference method, at 95% confidence limits. The developed procedure was cost-effective, simple, reliable and down-scaled and offered traceability as an alternative for the assay of carbaryl residues in herbal medicines.

## Figures and Tables

**Table 1:** Comparison of peroxidase enzymatic reaction-DLLME and smartphone-based digital image method with other spectrophotometric and digital image colorimetric methods for the determination of carbaryl in various samples.

Detection Technique	Pre-concentration Method	Reagent	Linearity	^a^ LOD	Recovery (%)	^b^ RSD (%)	Sample	Reference
Spectrophotometry	-	Diazotized 2-aminonaphthalenesulfonic acid	0.01–0.1 mg· L^−1^	-	96–98	-	Soil and insecticide	[32]
Spectrophotometry	-	p-Aminophenol, p-N,N-dimethylphenylenediamine, dihydrochloride, and 1-amino-2-naphthol-4-sulphonic acid	0.08–1 mg L^−1^	0.08 mg·L^−1^	92.0–97.5	1	Insecticide, water and grains	[35]
Spectrophotometry	-	2,6-Dibromo-4-methylaniline, 2,4,6-tribromoaniline, and 2,6-dibromo-4-nitroaniline	0.6–10.0 mg· L^−1^	0.825 mg·L^−1^	94.20–99.00	<2	Environmental Samples	[34]
Spectrophotometry	^c^ CPE	Rhodamine-B	0.04–0.4 mg· L^−1^	0.005 mg·L^−1^	97.80–101.20	<2	Water and grains	[36]
Spectrophotometry	^d^ DLME and ^e^ DMSPE	2-Naphthylamine-1-sulfonic acid	10–100 μg·L^−1^	8 ng·mL^−1^	97.3–108.1	8.5	Tap water, field water and fruit juice	[58]
Spectrophotometry	^f^ SPE ^g^Q uEChERS and ^h^ DLLME	4-AP, H_2_O_2_ with crude rubber tree bark peroxidase extracts	0.1–3.0 mg L^−1^	0.06 mg·L^−1^	83–118	<4	Vegetable sample	[52]
Digital image colorimetry	^i^ LPME	4-Methoxybenzene-diazonlum tetrafluoroborate (MBDF)	0.03–30.0 mg·kg^−1^	0.006–0.008 mg·kg^−1^	92.3–105.9	<5	Food sample	[41]
Smartphone-based digital image analysis	DLLME	4-AP, H_2_O_2_ with non-purified peroxidase extracts from Senna siamea Lam. bark	0.10-0.50 mg·L^−1^	0.03 mg·L^−1^	82.5–108.2	4.9	Pharmaceutical sample	This work

^a^ LOD is limit of detection; ^b^ RSD is relative standard deviation; ^c^ CPE is could point extraction; ^d^ DLME is dispersive liquid microextraction; ^e^ DMSPE is dispersive μ-solid phase extraction, ^f^ SPE is solid phase extraction; ^g^ QuEChERS is Quick, Easy, Cheap, Effective, Rugged and Safe; ^h^ DLLME is dispersive liquid-liquid microextraction and ^i^ LPME is liquid phase microextraction.

**Table 2:** Mean recovery percentage of spiked standard carbaryl into real samples and concentration of carbaryl residues in *Andrographis paniculate* herbal medicines obtained by smartphone-based digital images and HPLC-UV.

Sample	Added (mg·L^−1^)	Smartphone-Based Digital Images (*n* = 3)			HPLC-UV (*n* = 3)		
		Found (mg·L^−1^ ± SD)	Mean Recovery, %(RSD)	Carbaryl Content (mg·kg^−1^ ± SD)	Found (mg·L^−1^ ± SD)	Mean Recovery, %(RSD)	Carbaryl Content (mg·kg^−1^ ± SD)
1	0.1	0.1_1_ * ± 0.0_1_	108(6)	9.48 ± 0.15	0.100 ± 0.005	100(7)	9.72 ± 0.30
	0.2	0.2_0_ ± 0.0_1_	100(2)		0.177 ± 0.009	88(5)	
	0.3	0.3_1_ ± 0.0_1_	104(9)		0.289 ± 0.014	96(5)	
2	0.1	0.1_0_ ± 0.0_1_	101(10)	<LOD	0.100 ± 0.005	100(2)	<LOD
	0.2	0.2_0_ ± 0.0_1_	99(3)		0.184 ± 0.009	93(5)	
	0.3	0.2_5_ ± 0.0_1_	84(5)		0.340 ± 0.010	114(3)	
3	0.1	0.0_9_ ± 0.0_1_	87(3)	<LOD	0.100 ± 0.003	100(1)	<LOD
	0.2	0.1_9_ ± 0.0_0_	92(2)		0.189 ± 0.004	95(2)	
	0.3	0.3_0_ ± 0.0_1_	101(2)		0.288 ± 0.011	96(3)	
4	0.1	0.1_0_ ± 0.0_0_	98(5)	13.55 ± 0.34	0.089 ± 0.004	89(3)	14.83 ± 0.13
	0.2	0.2_0_ ± 0.0_0_	98(4)		0.198 ± 0.012	99(6)	
	0.3	0.3_2_ ± 0.0_0_	105(5)		0.285 ± 0.009	95(3)	
5	0.1	0.0_8_ ± 0.0_0_	83(6)	6.98 ± 0.16	0.097 ± 0.007	97(5)	6.57 ± 0.11
	0.2	0.2_0_ ± 0.0_0_	98(3)		0.184 ± 0.013	92(7)	
	0.3	0.2_7_ ± 0.0_0_	91(4)		0.284 ± 0.009	94(3)	
6	0.1	0.1_0_ ± 0.0_0_	103(4)	16.22 ± 0.29	0.095 ± 0.008	95(6)	15.56 ± 0.32
	0.2	0.2_1_ ± 0.0_1_	105(2)		0.193 ± 0.005	97(3)	
	0.3	0.3_2_ ± 0.0_0_	106(3)		0.282 ± 0.007	93(3)	
7	0.1	0.1_0_ ± 0.0_0_	101(5)	9.42 ± 0.97	0.102 ± 0.003	102(2)	10.15 ± 0.40
	0.2	0.2_0_ ± 0.0_0_	98(5)		0.195 ± 0.003	99(2)	
	0.3	0.2_6_ ± 0.0_0_	87(2)		0.280 ± 0.006	93(2)	
8	0.1	0.1_1_ ± 0.0_1_	109(5)	<LOD	0.103 ± 0.005	103(7)	<LOD
	0.2	0.2_0_ ± 0.0_0_	99(2)		0.190 ± 0.006	95(3)	
	0.3	0.2_9_ ± 0.0_1_	96(4)		0.307 ± 0.003	102(2)	
9	0.1	0.0_9_ ± 0.0_1_	90(5)	<LOD	0.104 ± 0.003	103(2)	<LOD
	0.2	0.1_9_ ± 0.0_1_	93(4)		0.212 ± 0.009	106(5)	
	0.3	0.2_7_ ± 0.0_1_	91(3)		0.315 ± 0.006	105(2)	
10	0.1	0.1_1_ ± 0.0_0_	108(4)	5.54 ± 0.13	0.103 ± 0.003	103(3)	6.31 ± 0.70
	0.2	0.1_9_ ± 0.0_1_	93(5)		0.195 ± 0.002	103(3)	
	0.3	0.2_9_ ± 0.0_1_	95(3)		0.310 ± 0.006	103(3)	

* The subscripts are the second decimal place.

## Data Availability

Not applicable.

## Supplementary Materials

The following supporting information can be downloaded at: https://www.mdpi.com/article/10.3390/molecules27103261/s1, Figure S1: Light control box for smartphone-based digital imaging for the determination of carbaryl, Figure S2: RGB profile plots of color intensities obtained from smartphone-based digital imaging in the enzymatic reaction and DLLME in a light control box for carbaryl in the range 0–0.50 mg·L^−1^, Figure S3: Plots of intensity difference (delta intensity) versus carbaryl concentration: (a) delta red intensity (b) delta green intensity, and (c) delta blue intensity, (delta intensity being the intensity due to that carbaryl concentration subtracted by that of blank), Table S1: Summarized selected conditions of smartphone-based digital images with DLLME for the determination of carbaryl residues.

## Sample Availability

Strains are available from the authors.
