# Supplementary material for: A Simple and Reliable Dispersive Liquid-Liquid Microextraction with Smartphone-Based Digital Images for Determination of Carbaryl Residues in Andrographis paniculata Herbal Medicines Using Simple Peroxidase Extract from Senna siamea Lam. Bark
Source: Molecules. 2022 May 19;27(10):3261. doi: 10.3390/molecules27103261 (PMC9147045; doi:10.3390/molecules27103261)
Supplement: Supplementary file 1 [file molecules-27-03261-s001.zip › molecules-1691116-supplementary.pdf]

molecules

MDPI

Supplementary MAterial

A Simple and Reliable Dispersive Liquid-Liquid Microextraction with Smartphone-Based Digital Images for Determination of Carbaryl Residues in *Andrographis paniculata* Herbal Medicines Using Simple Peroxidase Extract from *Senna siamea* Lam. Bark

Sam-ang Supharoek [1,2], Watsaka Siriangkhawut [3], Kate Grudpan [4] and Kraingkrai Ponhong [3,*]

[1] Department of Chemistry and Center of Excellence for Innovation in Chemistry, Faculty of Science, Mahidol University, Bangkok 10400, Thailand; samang.sup@mahidol.ac.th
[2] Department of Medical Science, Mahidol University, Amnatcharoen Campus, Amnat Charoen 37000, Thailand
[3] Creative Chemistry and Innovation Research Unit, Department of Chemistry and Center of Excellence for Innovation in Chemistry, Faculty of Science, Mahasarakham University, Maha Sarakham 44150, Thailand; watsaka@hotmail.com
[4] Department of Chemistry, Faculty of Science and Center of Excellence for Innovation in Analytical Science and Technology for Biodiversity-based Economic and Society, Chiang Mai University, Chiang Mai 50200, Thailand; kgrudpan@gmail.com
* Correspondence: kraingkrai.p@msu.ac.th; Tel.: +66-43754-246

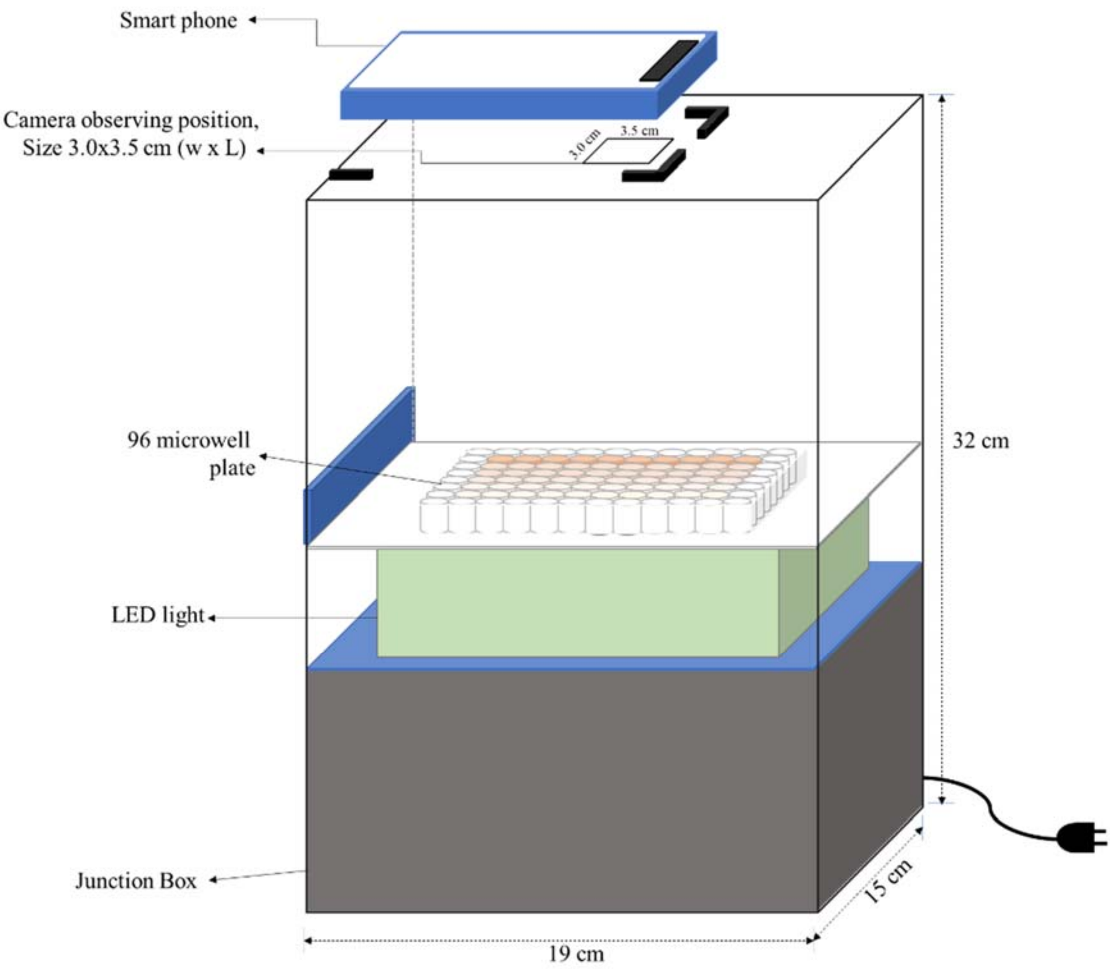

Figure S1. Light control box for smartphone-based digital imaging for the determination of carbaryl.

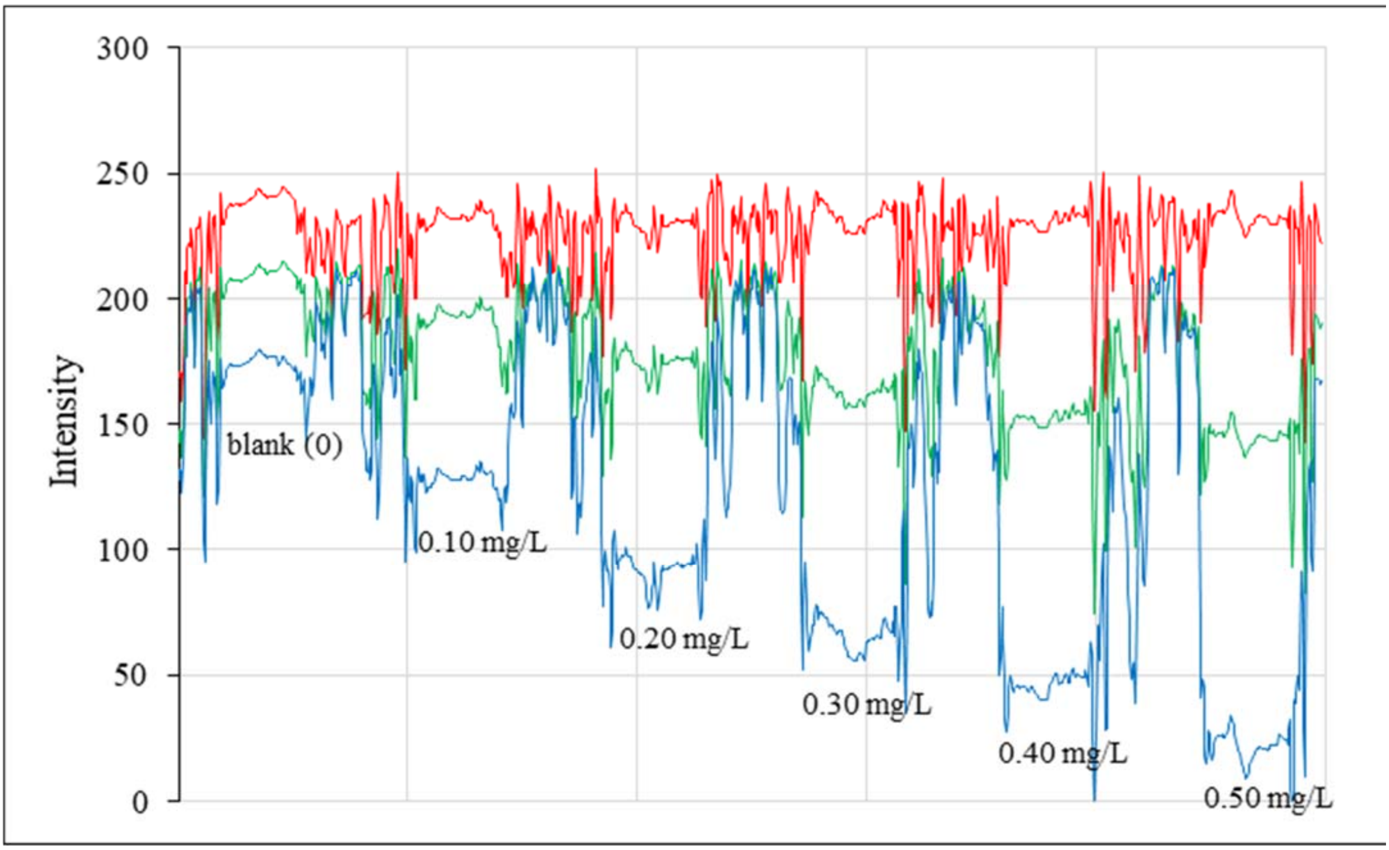

Figure S2. RGB profile plots of color intensities obtained from smartphone-based digital imaging in the enzymatic reaction and DLLME in a light control box for carbaryl in the range 0–0.50 mg·L^{-1}.

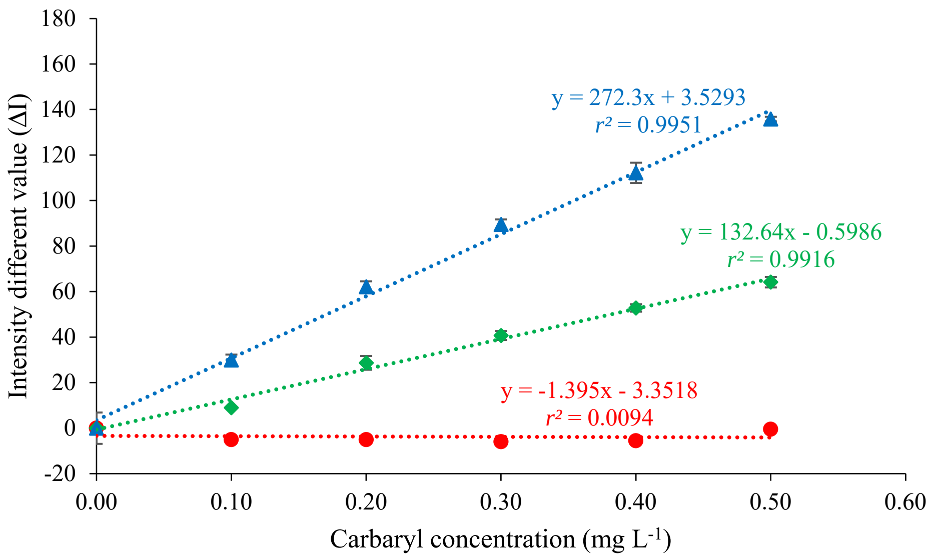

Figure S3. Plots of intensity difference (delta intensity) versus carbaryl concentration: (a) delta red intensity (b) delta green intensity, and (c) delta blue intensity, (delta intensity being the intensity due to that carbaryl concentration subtracted by that of blank).

Table S1. Summarized selected conditions of smartphone-based digital images with DLLME for the determination of carbaryl residues.

Parameter	Studied Condition	Selected Condition
pH	3–7	6
4-aminoantipyrine concentration (mg·L^{-1})	50–200	150
Hydrogen peroxide concentration (mmol·L^{-1})	0.01–1.0	0.3
Volume of enzyme (µL)	10–200	150
Incubation time (min)	1–20	10
Type of extraction solvent	Chloroform, dichloromethane, octanol and 1-dodecanol	Dichloromethane
Volume of extraction solvent (µL)	100–700	500
Type of dispersive solvent	Acetonitrile, ethanol, methanol and acetone	Ethanol
Volume of dispersive solvent (µL)	100–700	300
Ionic strength (%*w*/*v*)	0.6–1.4	1.0
Vortex time (min)	0.1–3	1
Centrifugation time (min)	1–10	7
